# Enhancing Mushroom Growing Substrate Through Biochar and Agro-Waste Integration to Promote Overall Productivity and Economic Impact in *Lentinus squarrosulus* Cultivation

**DOI:** 10.3390/jof12080590

**Published:** 2026-08-09

**Authors:** Worawoot Aiduang, Orlavanh Xayyavong, Kritsana Jatuwong, Chawonplusz Suppsorranna, Tanongkiat Kiatsiriroat, Wassana Kamopas, Saisamorn Lumyong

**Affiliations:** 1 Office of Research Administration, Chiang Mai University, Chiang Mai 50200, Thailand; worawoot.aiduang@cmu.ac.th (W.A.); kritsana.ja@cmu.ac.th (K.J.); 2 Department of Biology, Faculty of Science, Chiang Mai University, Chiang Mai 50200, Thailand; xorlavanh@yahoo.com; 3 Department of Biology, Faculty of Science, Champasack University, Pakse 16010, Laos; 4 Bachelor of Arts (Communication for Economics) College of Social Communication Innovation (COSCI), Srinakharinwirot University (SWU), Bangkok 10110, Thailand; plearn@agera-bioactives.com; 5 Department of Mechanical Engineering, Faculty of Engineering, Chiang Mai University, Chiang Mai 50200, Thailand; tanongkiat_k@yahoo.com; 6 Multidisciplinary Research Institute, Chiang Mai University, Chiang Mai 50200, Thailand; wassana.kamopas@cmu.ac.th; 7 Center of Excellence in Microbial Diversity and Sustainable Utilization, Chiang Mai University, Chiang Mai 50200, Thailand

**Keywords:** biochar application, biomass valorization, mushroom cultivation, *Lentinus* mushroom, SDGs 9

## Abstract

This study explores the potential of integrating agro-wastes with bamboo biochar to develop optimized substrates for cultivating *Lentinus squarrosulus*, aiming to enhance mycelial growth, productivity, nutritional quality, and economic returns. The chemical characterization revealed clear contrasts between sawdust and corn dust, where sawdust provided a carbon-rich but nitrogen-limited structure, while corn dust offered a greater nutrient availability. When formulated into mixed substrates, particularly at a 50:50 ratio, a more balanced physicochemical profile was achieved, improving substrate suitability for fungal growth. The incorporation of biochar (1–10%) further enhanced substrate performance by improving porosity, nutrient retention, and pH stability. Among treatments, mixed substrates supplemented with 5–10% biochar exhibited the highest mycelial growth rates and dense colonization, significantly reducing contamination rates. These conditions facilitated production cycles, with earlier primordia formation and harvesting compared to single-substrate systems. Yield performance was markedly improved in mixed formulations, especially those containing 1–5% biochar, which produced the highest fruiting body numbers, weights, and total yields. Biological efficiency exceeded 99% in optimal treatments, indicating highly efficient substrate conversion. Nutritional analysis demonstrated that substrate composition also influenced mushroom quality, with corn dust enhancing protein content, while mixed substrates promoted balanced fiber, carbohydrate, and energy profiles. Economic evaluation confirmed that integrating agro-wastes with moderate biochar levels significantly increased profitability, with the 50:50 sawdust–corn dust formulation supplemented with 5% biochar yielding the highest returns. Overall, this study highlights that strategic substrate design, balancing lignocellulosic structure, nutrient availability, and physicochemical properties, can substantially improve mushroom productivity and economic viability.

## 1. Introduction

Mushroom cultivation plays an important role in sustainable agriculture practice due to its ability to convert agricultural waste into valuable food products [1]. Various lignocellulosic materials such as sawdust and corn residues have been widely used as substrates for mushroom production. However, each substrate presents limitations in terms of nutrient availability and physical structure, which can affect mushroom yield and biological efficiency [2]. Conventional substrates, commonly based on industrial by-products, especially sawdust, frequently face challenges related to a shortage of raw materials, a significant risk of microbial contamination, and rising manufacturing costs [3,4]. Limitations highlight the need for different, low-cost, and sustainable substrate formulations that enhance mycelial growth, reduce contamination, and improve overall yield and product quality [5].

Agro-waste materials offer significant potential as sustainable substrates for mushroom cultivation, providing a low-cost, eco-friendly alternative while enhancing resource efficiency and supporting large-scale production potential [6]. Among these, corn dust, a fine by-product generated during corn processing, is abundant, low-cost, and rich in lignocellulosic compounds that serve as an effective carbon source for fungal growth [7,8]. Produced during mechanical corn shelling or milling, corn dust accounts for approximately 2% of the total corn processed. In recent years, it has attracted increasing interest as a feed ingredient for ruminants and as a potential bioenergy resource due to its availability and nutritional composition. However, its utilization remains limited by its relatively high fiber content and fine particle size [9]. When used as the sole substrate component, corn dust tends to compact easily, reducing substrate porosity and aeration while increasing moisture retention and the risk of microbial contamination [10]. These unfavorable physical properties can restrict mycelial colonization and ultimately reduce cultivation performance. Therefore, strategic modification of corn dust-based substrates is essential to improve their physical structure and chemical balance. Optimizing mixing ratios with complementary materials can enhance porosity, moisture regulation, and overall substrate stability, thereby supporting efficient and sustainable mushroom production [11].

Biochar, a carbon-rich material produced through pyrolysis, has been reported to improve substrate aeration, water retention, and nutrient availability. These properties may enhance mycelial growth and fruiting performance in mushroom cultivation [12,13]. Among various biochar types, bamboo biochar is particularly attractive because it is derived from renewable bamboo residues that are abundantly available, contain valuable mineral nutrients, and widely generated in industrial and agricultural sectors across many tropical and subtropical regions. However, these bamboo recyclables are often underutilized, leading to challenges associated with carbon management and missed opportunities for sustainable resource utilization [14]. When utilized in growing media, biochar can improve porosity, water-holding capacity, and nutrient retention, while also facilitating effective airflow during pasteurization, thereby improving the elimination of undesirable microorganisms [15]. In addition, recent studies indicate that biochar can improve microbial proliferation, stimulate fungal colonization, and enhance overall crop performance. By creating a more porous and oxygen-rich environment, biochar not only improves moisture management but also increases cation exchange capacity over extended periods [16,17]. These characteristics highlight its strong potential as a valuable component in mushroom substrate formulations.

Although previous studies have explored the use of individual agro-waste materials, limited research has focused on the combined use of mixed substrates integrated with biochar and their impact on both productivity and economic profitability. Furthermore, there is a lack of comprehensive evaluation linking substrate formulation, biological performance, and economic return, particularly under practical cultivation conditions. The integration of corn dust agro-waste into a primary sawdust-based substrate, combined with bamboo biochar, may therefore offer a synergistic strategy for developing high-performance mushroom growing media. This approach is expected to promote rapid and uniform mycelial expansion, reduce contamination during spawn preparation and cultivation, and ultimately improve mushroom yield and nutritional quality. Earlier research studies suggest that incorporating agricultural waste materials into sawdust at ratios of approximately 25–50% can enhance mycelial growth and production outcomes [18,19,20]. Similarly, the addition of biochar at levels of about 1–10% of the substrate has been reported to improve cultivation efficiency across several mushroom species [13,17,20,21,22,23]. Beyond biological benefits, the use of locally available agro-waste and biochar supports circular economy principles by minimizing waste streams and lowering production costs for small- to large-scale cultivators [24]. 

Therefore, this study aims to evaluate the effects of different substrate compositions, including sawdust, corn dust, and varying levels of biochar, on mushroom growth, yield, and economic profitability, with a particular focus on *L. squarrosulus*, an economically important species widely cultivated in tropical and subtropical regions. *Lentinus squarrosulus* is a xylotrophic white-rot basidiomycete that efficiently colonizes and degrades lignocellulosic biomass through the production of ligninolytic enzymes, making it well suited for cultivation on a wide range of agricultural and forestry residues that serve as nutrient-rich substrates for fungal growth [25]. The research evaluates the overall physicochemical properties of the formulated substrates and examines their effects on mycelial growth, spawn quality, contamination incidence, and mushroom yield under small-scale greenhouse conditions. In addition, the nutritional composition of the harvested mushrooms was analyzed to assess product quality. Furthermore, a primary economic analysis was conducted to determine the cost-efficiency and practical feasibility of the proposed substrate formulations. By systematically integrating biological, chemical, and economic evaluations, this work seeks to provide a scientifically grounded and scalable strategy for enhancing mushroom growing media through biochar and agro-waste integration.

## 2. Materials and Methods

**Figure 1:**
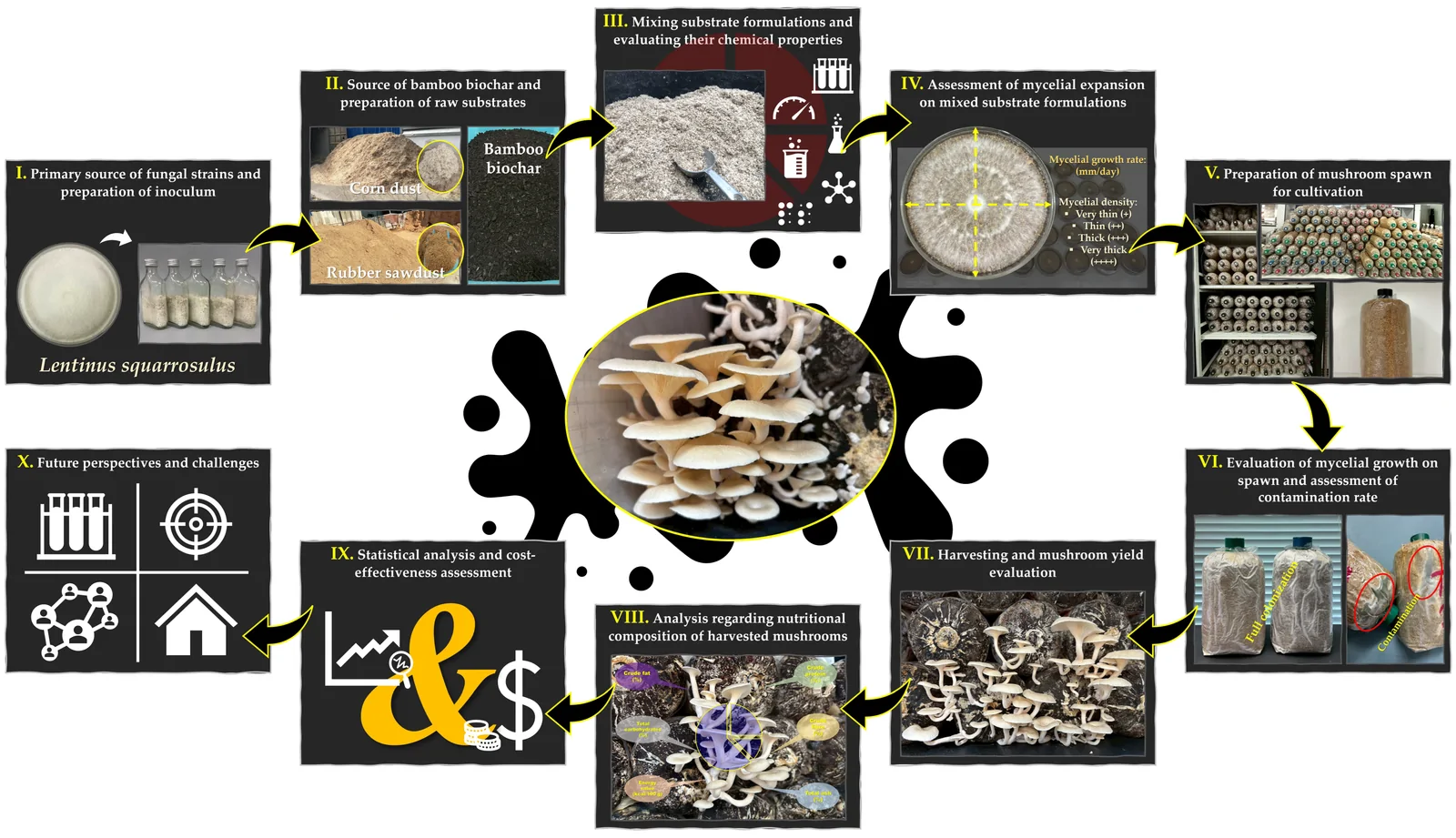
Schematic overview of the experimental workflow used to evaluate the potential of *L. squarrosulus* for sustainable mushroom production through the utilization of agro-industrial residues supplemented with bamboo biochar-based substrate formulations.

The experimental workflow was designed to evaluate the potential of *L. squarrosulus* for sustainable mushroom production using agro-industrial residues and bamboo biochar-based substrates. This investigation covered fungal inoculum preparation, substrate formulation, physicochemical characterization, and assessment of mycelial growth performance. Selected substrate mixtures were further evaluated for spawn production, cultivation efficiency, mushroom yield, nutritional composition, and production cost-effectiveness. The overall methodology is summarized in Figure 1, illustrating the integrated approach from fungal culture preparation to future application potential.

### 2.1. Primary Source of Fungal Strains and Preparation of Inoculum

A pure culture of *L. squarrosulus* CMU-WE001 was obtained from the Saisamorn Lumyoung Laboratory Culture Collection (SLLC), Faculty of Science, Chiang Mai University, Thailand, and used throughout this study. Grain spawn was prepared using sorghum grains as the carrier substrate following the method described by Aiduang et al. [26]. The prepared grain spawn was subsequently used as the inoculum for all experiments.

### 2.2. Source of Bamboo Biochar and Preparation of Raw Substrates

The biochar used in this study was derived from bamboo and produced via a slow pyrolysis process, following the conditions described in our previous research (500 °C for 2 h) as reported by Jatuwong et al. [27]. According to that study, the resulting biochar exhibited favorable physicochemical properties, including a porous structure, high carbon content, alkaline pH, and good water-holding capacity, making it a suitable supplement for mushroom cultivation substrates. After production, the biochar was ground into a fine powder and sieved through a 2 mm mesh to obtain a uniform particle size prior to use.

The main substrates used in this study comprised two types of agro-industrial wastes: rubber sawdust and corn dust. These materials were obtained from a local sawmill and a corn processing factory in Chiang Mai Province, Thailand. Prior to experimentation, the substrates were sieved using a rotating separator equipped with a 9-mm mesh to remove any remaining contaminants and to obtain particles of comparable size for subsequent analyses.

### 2.3. Mixing Substrate Formulations and Evaluating Their Chemical Properties

The chemical properties of each raw substrate were analyzed prior to use applying standardized analytical methods. The main chemical components of the raw materials, including cellulose, hemicellulose, and lignin contents, were determined by detergent fiber analysis following the methods of the Association of Official Analytical Chemists (AOAC). Rubber sawdust (RS) and corn dust (CD) were then mixed with supplementary ingredients (SI) consisting of 5% rice bran, 1% calcium carbonate, 2% calcium sulfate, and 0.2% sodium sulfate (dry weight basis), followed by the addition of varying levels of bamboo biochar (BB-Char) according to the formulations presented in Table 1. Each formulated substrate was dried at 60 °C for 24 h to achieve a hygroscopic moisture content of approximately 8.0 ± 0.5%, and subsequently ground to a fine particle size (<2 mm) before further analysis [28].

Electrical conductivity (EC) and pH were measured using a 1:10 (*w*/*v*) substrate-to-distilled water suspension. The EC was determined with an Ecoscan COND 6+ Conductivity Meter (EUTECH Instruments, Melaka, Malaysia), while pH was measured using a Sartorius PB-10 pH meter (Sartorius, Göttingen, Germany). Organic carbon (OC) was analyzed using the Walkley and Black method (1934), and organic matter (OM) content was calculated by multiplying the OC percentage by the appropriate conversion factor. Total nitrogen (N) content was determined using the Kjeldahl method, and the C/N ratio was subsequently calculated. All analyses were conducted at the Agricultural Technology Services Center, Faculty of Agriculture, Chiang Mai University, Chiang Mai, Thailand. Each treatment was analyzed in triplicate to confirm data reliability.

### 2.4. Assessment of Mycelial Expansion on Mixed Substrate Formulations

Each substrate formulation described in Section 2.3 (Table 1) was prepared to evaluate mycelial expansion under controlled conditions. The moisture content of the mixtures was adjusted to 60–70% by adding distilled water. Subsequently, 10 g of each substrate formulation was put to a 90 mm-diameter Petri dish and sterilized for 60 min at 121 °C. After cooling to room temperature, a 10 mm diameter mycelial disk of *L. squarrosulus* was aseptically placed at the center of each plate. The plates were then incubated at 30 °C for 14 days [26].

Mycelial growth was recorded daily by measuring colony diameters along two perpendicular axes. The radial growth rate (mm/day) was calculated from the increase in the average colony diameter over time. Mycelial density was visually assessed on day 7 and at the end of the incubation period and classified as very thin (+; score 1), thin (++; score 2), thick (+++; score 3), or very thick (++++; score 4) according to the adapted criteria described by De Leon et al. [29]. Each treatment was performed with five replicates.

### 2.5. Mushroom Spawn Preparation, Mycelial Colonization, and Contamination Assessment

The dried substrates prepared according to the formulations described in Section 2.3 were adjusted to a final moisture content of approximately 60–70%. A total of 600 g of the prepared substrate was then packed into polypropylene (PP) bags (3.5 × 11.5 in). Each bag was fitted with a PP pipe ring and sealed with a cotton-plastic plug. The bags were sterilized in an autoclave at 121 °C for 60 min and subsequently allowed to cool to room temperature for 24 h. After cooling, each bag was inoculated with 6 g of mycelial inoculum, corresponding to a substrate-to-inoculum ratio of 100:1 (*w*/*w*). The inoculated bags were incubated in a dark incubation room at ambient temperature (24–36 °C) until the mushroom mycelium completely colonized the substrate and was ready for fruiting body induction [26]. Thirty replicates were prepared for each experimental treatment in this study.

Following incubation, mycelial growth on the spawn of each treatment was evaluated by recording the time required for complete substrate colonization, along with the contamination rate. Vegetative growth was determined through direct visual observation until full mycelial colonization of the substrates by *L. squarrosulus* was achieved. The appearance of contaminants, particularly green mold across the genus *Trichoderma*, was carefully observed and recorded [30].

Once full colonization was confirmed, all substrate bags were transferred to a small-scale greenhouse and arranged horizontally on cultivation shelves. On the first day, the PP pipe ring of each spawn bag was removed, and the bag opening was carefully pulled apart to expose the colonized substrate at the front. On the second day, a slit was carefully made at the neck of each bag, and the plastic wrap was removed to stimulate fruiting body development. Moisture management began on day three, with watering applied to maintain suitable humidity levels [18]. The timing of primordia formation and the appearance of the first harvestable fruiting bodies were recorded after induction to assess cultivation performance [31].

### 2.6. Harvesting and Mushroom Yield Evaluation

For mushroom production, 24 fully colonized cultivation bags for each substrate formulation were selected and continuously monitored for yield assessment over a period of approximately 12 weeks. During the experimental period (August–November 2025), the study was conducted in the rainy season, when ambient humidity was naturally high. Greenhouse relative humidity was maintained at approximately 75–85% with watering once daily.

Once fruiting bodies appeared, mushrooms were harvested at the same time each day. The market quality of the harvested basidiocarps was evaluated following the criteria described by Chiejina and Osibe [31]. All harvested mushrooms were counted and weighed, with useful fruiting bodies typically exhibiting cap diameters of approximately 3–7 cm for each treatment. Fresh samples were then oven-dried at 60 °C for 24–48 h until a constant weight was achieved, and the dry weight was recorded for each harvest.

Total yield (g per bag) and the number of flushes for each treatment were recorded throughout the cultivation period. In addition, biological efficiency (BE) was calculated to evaluate production performance using the following formula: BE (%) = Fresh weight of mushrooms harvested (g)/Dry weight of substrate (g) × 100.

### 2.7. Analysis Regarding Nutritional Composition of Harvested Mushrooms

After oven drying at 60 °C for 24–48 h, mushrooms harvested from the different substrate formulations were subjected to proximate analysis to determine their nutritional composition. Analyses of crude protein, crude fat, ash, and crude fiber were conducted following standard methods of the Association of Official Analytical Chemists (AOAC). Crude protein content was calculated using a nitrogen-to-protein conversion factor of 4.38. Total carbohydrate content was determined by difference using the formula: Total carbohydrates (%) = 100 − (% moisture + % crude protein + % crude fat + % crude fiber + % ash). 

The energy value (EV) of the harvested mushroom samples was determined using bomb calorimetry according to the AOAC standard method for food energy analysis. All analyses were performed at the Central Laboratory and the Animal Nutrition Laboratory, Department of Animal and Aquatic Sciences, Faculty of Agriculture, Chiang Mai University, Thailand.

### 2.8. Statistical Analysis and Cost-Effectiveness Assessment

Statistical analyses were performed using one-way analysis of variance (ANOVA) with SPSS software, version 17.0 (SPSS Inc., Chicago, IL, USA). Differences among mean values were assessed using Duncan’s multiple range test at a significance level of *p* ≤ 0.05. In addition, production costs and potential future challenges were evaluated through comparisons with conventional practices, providing information regarding their economics, performance, and future development opportunities.

## 3. Results and Discussion

### 3.1. Chemical Properties of Raw Substrates and Mixed Formulations

The chemical composition of the primary substrates and their modified formulations played a decisive role in shaping both mycelial growth and yield performance of *L. squarrosulus*. Clear contrasts were observed among the sawdust-based, corn dust-based, and mixed-substrate formulations, demonstrating differences in lignocellulosic structure, nutrient availability, and physicochemical balance (Table 2).

Sawdust, characterized by high cellulose (50.54%) and lignin (18.09%) but very low nitrogen (0.22%), exhibited an extremely high C/N ratio (258.12), indicating a carbon-rich but nutrient-limited substrate. This condition typically limits rapid mycelial expansion due to insufficient nitrogen for protein synthesis, despite providing strong structural support for prolonged growth [32]. In contrast, corn dust contained significantly higher hemicellulose (48.99%) and nitrogen (0.91%), with a much lower C/N ratio (58.29), making it more readily degradable and nutritionally accessible for fungal metabolism. This composition also supports earlier development and prolonged fruiting periods [33]. However, the relatively high EC (13.81 mS/cm) may impose natural and chemical challenges on mycelial activity [34]. Meanwhile, the pH of the mixed substrate formulations ranged from 5.75 to 6.60, which falls within the suitable range (5.0–8.0) for *L. squarrosulus*, with optimal growth typically observed near pH 6.5–7.0 [35]. 

**Table 2:** Chemical composition of the primary raw substrates and the properties of mixed substrate formulations for each treatment in this study.

Substrates	Treatment	Cellulose (%)	Hemicellulose (%)	Lignin (%)	EC (mS/cm)	pH	OC (%)	N (%)	OM (%)	C/N
Raw substrates	Sawdust—pure substrate	50.54 ± 0.63	13.80 ± 0.76	18.09 ± 0.72	5.37 ± 0.14	7.29 ± 0.04	57.12 ± 0.73	0.22 ± 0.03	98.48 ± 1.26	258.12 ± 31.36
	Corn dust—pure substrate	30.71 ± 1.01	48.99 ± 0.22	3.84 ± 0.23	13.81 ± 2.11	5.79 ± 0.03	53.22 ± 1.08	0.91 ± 0.01	91.76 ± 1.86	58.29 ± 1.75
	Bamboo biochar [27,36]	-	-	-	11.33 ± 0.05	8.83 ± 0.03	64.4–86.3	0.87–1.10	-	74.02–78.45
Mixed substrate formulations	T1-100S + SI	-	-	-	15.12 ± 0.22 ^c^	6.60 ± 0.05 ^a^	54.52 ± 0.10 ^a^	0.24 ± 0.01 ^f^	93.99 ± 0.18 ^a^	224.14 ± 5.64 ^a^
	T2-100CD + SI	-	-	-	21.16 ± 2.06 ^a^	5.82 ± 0.10 ^ef^	51.71 ± 1.61 ^bcd^	0.94 ± 0.01 ^a^	89.15 ± 2.77 ^bcd^	54.82 ± 1.75 ^c^
	T3-100CD + SI + 1BB-Char	-	-	-	22.0 ± 2.39 ^a^	5.75 ± 0.05 ^f^	51.58 ± 0.89 ^bcd^	0.94 ± 0.01 ^a^	88.93 ± 1.53 ^bcd^	54.68 ± 0.97 ^c^
	T4-100CD + SI + 5BB-Char	-	-	-	21.49 ± 2.77 ^a^	5.77 ± 0.07 ^ef^	50.70 ± 1.01 ^d^	0.89 ± 0.02 ^b^	87.41 ± 1.74 ^d^	56.98 ± 1.03 ^c^
	T5-100CD + SI + 10BB-Char	-	-	-	19.87 ± 1.94 ^ab^	5.87 ± 0.04 ^de^	48.19 ± 1.10 ^e^	0.85 ± 0.02 ^c^	83.08 ± 1.90 ^e^	56.48 ± 1.18 ^c^
	T6-50S:50CD + SI	-	-	-	18.15 ± 0.27 ^bc^	5.87 ± 0.07 ^de^	54.09 ± 0.17 ^a^	0.60 ± 0.01 ^d^	93.25 ± 0.29 ^a^	89.66 ± 1.04 ^b^
	T7-50S:50CD + SI + 1BB-Char	-	-	-	16.61 ± 1.05 ^c^	5.95 ± 0.03 ^cd^	52.53 ± 0.82 ^b^	0.59 ± 0.02 ^d^	90.57 ± 1.41 ^b^	89.10 ± 3.39 ^b^
	T8-50S:50CD + SI + 5BB-Char	-	-	-	17.69 ± 0.81 ^bc^	6.03 ± 0.02 ^c^	52.35 ± 0.10 ^bc^	0.56 ± 0.01 ^e^	90.25 ± 0.18 ^bc^	92.96 ± 2.00 ^b^
	T9-50S:50CD + SI + 10BB-Char	-	-	-	17.04 ± 0.26 ^bc^	6.14 ± 0.03 ^b^	50.83 ± 0.69 ^cd^	0.56 ± 0.01 ^e^	87.63 ± 1.19 ^cd^	91.32 ± 2.19 ^b^

**Note:** Values are expressed as mean ± standard deviation. Different letters (T1–T9) within the same column indicate significant differences according to Duncan’s multiple range test (*p* < 0.05). Bamboo biochar characteristics were referenced from our previous study [27] and supported by comprehensive findings from Chen et al. [36].

When formulated into cultivation substrates, these intrinsic differences became more pronounced. The T1 treatment (100% sawdust) retained a high organic matter content (93.99%) but low nitrogen (0.24%), resulting in the highest C/N ratio (224.14). This condition supported normal but relatively slower mycelial growth and spawn running, leading to acceptable yet moderate yields [11]. Conversely, T2–T5 (100% corn dust with varying biochar levels) showed improved nitrogen availability (0.85–0.94%) and lower C/N ratios (54–57), which are generally advantageous for mycelial colonization. Most lignocellulosic substrates are inherently carbon-rich and nitrogen-deficient, typically ranging from 50:1 to 80:1, which exceeds the optimal range (20:1 to 40:1) required for efficient development of edible fungi [11,35]. Maintaining this balance is therefore crucial for optimal mycelial development. Although corn dust-based substrates exhibited improved C/N ratios, their relatively high electrical conductivity (19.87–22.0 mS/cm) may have affected overall mycelial growth and mushroom yield [37]. 

The integration of bamboo biochar further modified substrate properties in a dose-dependent practices. While biochar slightly reduced organic carbon and EC at higher application rates, it also improved pH buffering and nutrient retention. Notably, T5 (10% biochar) showed a reduction in EC and a more balanced chemical profile compared to lower biochar treatments, suggesting partial mitigation of salt stress. However, excessive biochar did not fully compensate for the limitations of corn dust alone.

The most favorable outcomes were observed in mixed substrates (T6–T9), where sawdust and corn dust were combined at a 50:50 ratio. These formulations achieved a more balanced nutrient profile, with moderate nitrogen content (0.56–0.60%), reduced EC (16.61–18.15 mS/cm), and middle C/N ratios (89–93). This balance may have supported both efficient enzymatic degradation and sustained mycelial growth [11]. Among these, T7 to T9 (with 1, 5 and 10% biochar) demonstrated interesting tendency physicochemical conditions, which might promote faster colonization, denser mycelial networks, and significantly higher yields. Overall, the findings suggest that neither high-carbon nor high-nitrogen substrates alone may not be optimal. In fact, an appropriate combination of lignocellulosic structure, nutrient availability, and controlled EC and pH is essential for optimizing fungal performance. The integration of agro-waste with biochar not only enhances substrate functionality but also provides a practical strategy for improving mushroom productivity while maintaining sustainability.

### 3.2. Mycelial Growth on Mixed Substrate Formulations

The mycelial growth rate and density of *L. squarrosulus* were significantly influenced by substrate composition and biochar supplementation (Table 3). Clear differences were observed among treatments, demonstrating that integrating agro-wastes with biochar markedly improved early vegetative development.

Among all treatments, T9 (50S:50CD + 10% BB-Char) achieved the highest mycelial growth rate at 16.81 mm/day, followed closely by T8 (50S:50CD + 5% BB-Char) at 16.19 mm/day. These values were significantly higher than the control T1 (100% rubber sawdust), which recorded 14.03 mm/day, and substantially greater than T2 (100% corn dust), which showed the slowest growth at 12.13 mm/day. The enhancement growth of more than 25% between T9 and T2 highlights the strong synergistic effect of combining substrates and enriching them with biochar.

Mycelial density followed a comparable variation. The highest density score (3.60) was recorded in T5, T8, and T9, indicating thick, compact, and rapid mycelial colonization. In contrast, T1 exhibited the lowest density (1.00), reflecting thin and less dense mycelial development despite its moderate radial growth rate. This suggests that efficient colonization depends on substrate accessibility and overall structural integrity rather than only growth rate.

**Figure 2:**
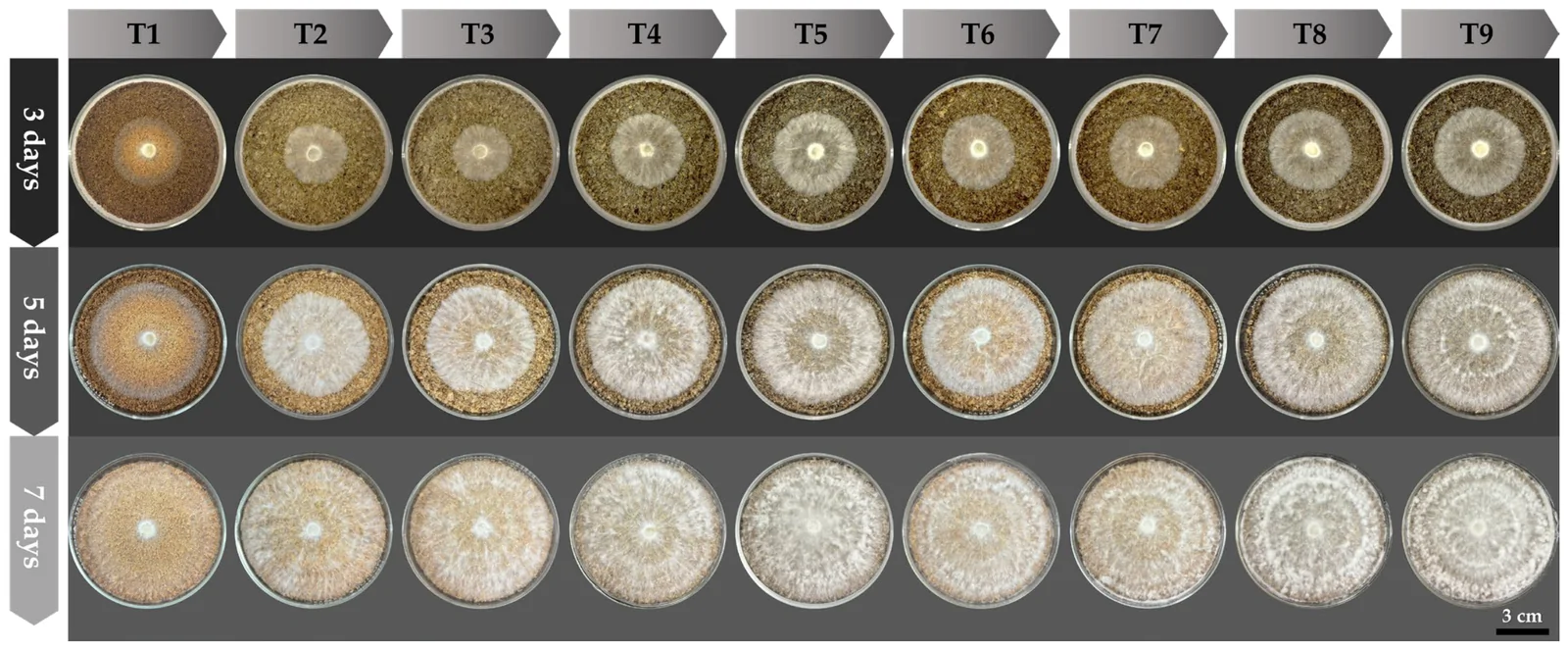
Mycelial expansion and density of *L. squarrosulus* cultivated on different mixed substrate formulations over a 7-day incubation period.

Visual observations over the 7-day incubation period (Figure 2) further confirmed these quantitative results. By the third day, early colonization was already more apparent in biochar-amended mixed substrates. On the fifth day, T8 and T9 exhibited nearly complete surface coverage with dense, white mycelium. By the seventh day, these treatments exhibited uniform and dense colonization across the entire substrate surface, whereas the pure corn dust or sawdust treatments showed slower growth and comparatively thinner mycelial coverage.

This finding aligns with previous studies indicating that greater substrate diversity improves nutrient availability and supports more stable microbial activity within the growing medium [32,38,39,40]. The enhanced performance observed in biochar-amended substrates can be attributed to improved physicochemical properties and increased nutrient accessibility. In particular, biochar likely enhanced substrate porosity and created favorable microenvironments that stimulated hyphal extension and branching [17,41]. Notably, while 10% biochar (T9) produced the fastest radial growth, 5% biochar (T8) achieved comparable density, suggesting that moderate supplementation may optimize both expansion speed and structural integrity. Overall, the integration of rubber sawdust and corn dust significantly enhanced mycelial performance compared to single-material substrates, particularly by promoting greater mycelial density. The addition of 5–10% biochar further accelerated growth and increased mycelial density, indicating improved substrate quality and colonization efficiency. These findings demonstrate that agro-waste integration combined with biochar enrichment provides a strong basis for rapid and strong mycelial establishment, ultimately supporting improved mushroom productivity in subsequent cultivation stages.

### 3.3. Mycelial Growth Performance and Contamination Rate of Mushroom Spawn

This investigation clearly demonstrates that integrating corn dust with biochar significantly enhances mushroom substrate performance, impacting mycelial growth, contaminant reduction, and fruiting development. Distinct differences among treatments reveal the importance of substrate composition in optimizing both biological effectiveness and production periods (Table 4).

Mycelial colonization was fastest in the mixed substrate treatments, particularly T9 (50S:50CD + 10% biochar) and T8 (50S:50CD + 5% biochar), which completed colonization within 26–28 days. In contrast, the 100% corn dust substrate (T2) required over 39 days, indicating slower mycelial expansion in single-component mixtures. The appropriate combination of rubber sawdust and corn dust potentially improved nutrient availability and substrate digestibility, leading to rapid vegetative growth [11,42]. The addition of biochar also raised colonization, potentially due to its highly porous structure, which improves water-holding capacity and aeration within the substrate. These physical properties, attributed to the complex pore architecture of biochar, create a more favorable microenvironment for mycelial development [41].

Contamination rates were markedly reduced by biochar addition. While the pure corn dust treatment exhibited the highest contamination (16.67%), biochar-added treatments, particularly at 5% and 10% levels, achieved minimal to zero contamination. This suggests that biochar may create a more stable and less favorable environment for competing microorganisms, thereby enhancing substrate condition and spawn quality. One possible explanation lies in the naturally alkaline nature of biochar. Its addition may increase the substrate pH, creating conditions that suppress common contaminants. This observation is consistent with the findings of Lombardi et al. [43], who reported that maintaining an alkaline substrate pH of 8–9 can effectively reduce the risk of *Trichoderma* infection. 

Fruiting development exhibited a comparable pattern in the biochar-amended treatments. The mixed substrate treatments, T6 and T7, achieved the shortest primordia formation period (approximately 49–50 days) and the earliest harvest (around 51 days). These were closely followed by T8 (50S:50CD + 5% BB-Char), which also demonstrated a relatively short primordia formation period and early harvest (about 52.07 and 53.60 days, respectively). In comparison, the 100% sawdust and 100% corn dust substrates required more than 59 and 84 days, respectively, to reach harvest, demonstrating significantly slower productive development. These results suggest that combining agro-wastes provides a greater appropriate mineral nutrient composition, facilitating a more efficient change from vegetative mycelial growth to yielding development [11]. Although higher biochar supplementation slightly prolonged the primordia formation period in some treatments, it consistently maintained low contamination levels and stable fruiting performance, indicating its supportive role in substrate quality and crop availability.

**Figure 3:**
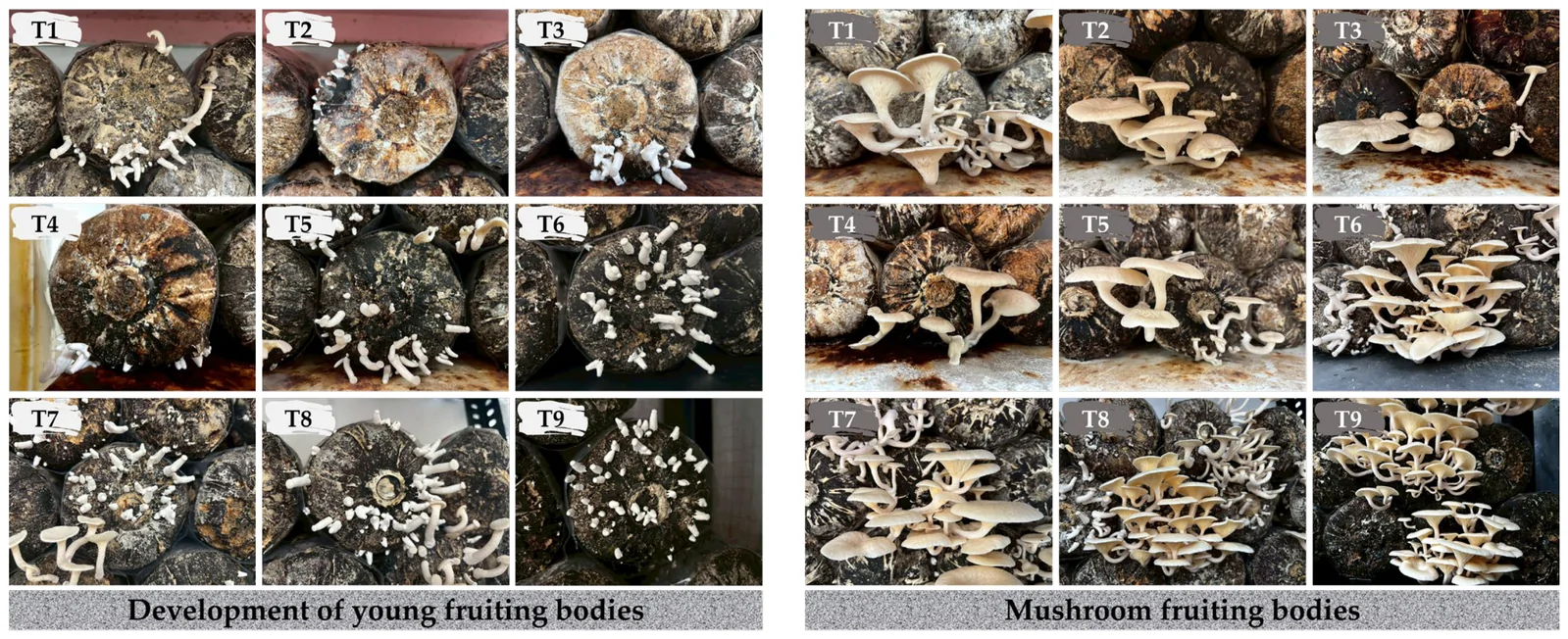
Development of young fruiting bodies and mature mushrooms observed across different substrate formulations in each treatment following bag opening.

Visual observations (Figure 3) supported these quantitative results. Treatments incorporating both corn dust mixtures and biochar produced more uniform primordia and well-developed fruiting bodies. The mushrooms appeared well-grown, with consistent mature form production and grouped growth, reflecting efficient substrate utilization. Overall, the integration of rubber sawdust, corn dust, and biochar improved biological performance while reducing contamination risks and shortening production cycles. Among the tested formulations, the 50S:50CD mixtures supplemented with minimum to moderate biochar levels (1–5%) proved particularly effective, providing an optimal balance between rapid mycelial colonization, early fruiting, and sustained substrate quality for *L. squarrosulus* cultivation.

### 3.4. Mushroom Yield and Biological Efficiency Across Different Substrate Formulations 

**Figure 4:**
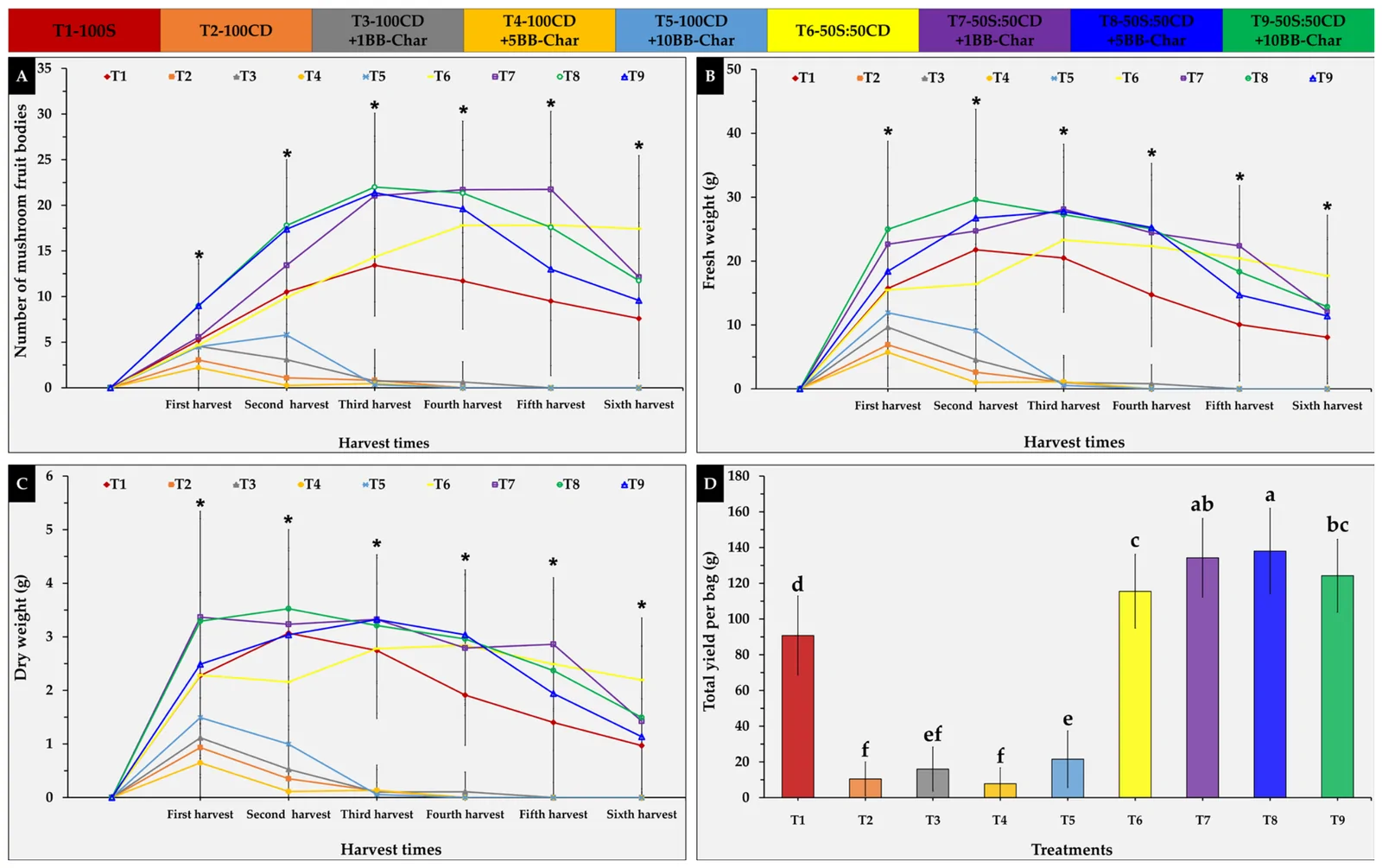
Overall mushroom yield across different substrate formulations: (**A**) number of fruiting bodies, (**B**) fresh weight, (**C**) dry weight, and (**D**) total yield per bag. Data are presented as mean ± standard deviation. Asterisks (*) indicate significant differences (*p* < 0.05) in Figure 4A–C, while different letters in Figure 4D denote significant differences among treatments according to Duncan’s multiple range test (*p* < 0.05).

The number of fruiting bodies, fresh weight, dry weight, and total yield per bag of mushrooms growing on various substrate formulations are all shown in Figure 4. The results reveal pronounced and statistically significant differences among treatments, clearly demonstrating the synergistic benefits of integrating agro-waste materials with biochar.

The results of this study demonstrated that substrate composition significantly influenced mushroom growth, yield, and economic efficiency. The mixed substrate (50% sawdust and 50% corn dust) showed superior performance compared to single substrates, indicating that a suitable mixture involving structural and nutritional components is essential for optimal mycelial development. In terms of fruiting body production (Figure 4A), T6, T7, T8, and T9 exhibited the largest and most sustained outputs across six harvests. Peak production occurred between the third and fourth harvests, where T7 and T8 reached approximately 21–22 fruit bodies per harvest, markedly higher than T1 (11–13 fruit bodies at peak) and significantly more than 100% corn dust treatments (T2–T5), which rarely exceeded five fruit bodies and showed no further production after early flushes.

Fresh weight in each flush (Figure 4B) exhibited a similar trend. T8 achieved the highest fresh weight during peak harvest, reaching nearly 30 g per flush, closely followed by T7 and T9 (approximate 27–28 g). In contrast, T2 and T4 recorded minimal fresh weights (below 7 g at peak), confirming the poor productivity of pure corn dust substrates even when amended with biochar. Similarly, dry weight results (Figure 4C) further reinforced these findings. Mixed treatments, particularly T7 and T8, attained maximum dry weights of approximately 3.2–3.5 g per flush, indicating efficient biomass accumulation and better substrate-to-fruit conversion. Meanwhile, single corn dust treatments remained below 1 g dry weight in most harvests.

The cumulative effect of these differences is clearly reflected in total yield per bag (Figure 4D). T8 produced the highest overall yield at approximately 138 g per bag, followed by T7 (around 134 g) and T9 (around 124 g). These values were significantly higher than T6 (around 115 g) and far exceeded T1 (around 90 g). The lowest yields were observed in T2 and T4, both below 11 g per bag.

Importantly, biochar supplementation at 1 and 5% within mixed substrates (T7 and T8) delivered optimal performance. Although 10% biochar (T9) maintained high productivity, yield slightly declined compared to 5%, suggesting that excessive biochar may alter nutrient balance or reduce substrate density. These findings are consistent with previous studies reporting that higher biochar incorporation levels can negatively affect mycelial growth and overall productivity in various mushroom species. This effect is likely associated with changes in the physicochemical properties of the substrate formulation, such as moisture content, pH, porosity, and nutrient availability, which directly influence mycelial growth and mushroom yield [13,21,44,45]. Overall, the integration of rubber sawdust and corn dust, particularly when supplemented with 1–5% biochar, significantly improved fruiting body formation, biomass accumulation, and total yield stability across multiple harvest cycles. This enhancement is largely attributed to the porous structure of biochar, which improves aeration and water retention, thereby creating a more favorable environment for mycelial growth and colonization. These results underscore the importance of substrate optimization as a key strategy for maximizing mushroom productivity while supporting the sustainable use of agro-waste resources.

Moreover, the results clearly demonstrate that integrating biochar with corn dust substrates substantially improves mushroom production, particularly in terms involving biological performance and flushing count (Table 5). Significant variation was observed among treatments in both number of flushes and BE, highlighting the importance of substrate composition in determining cultivation outcome. Among single-substrate treatments, 100% rubber sawdust (T1) performed relatively well, producing over five flushes with a BE of 54.97%. In contrast, 100% corn dust (T2) showed very poor productivity, with minimal flushes (1.13) and extremely low BE (8.68%), indicating that corn dust alone lacks the structural and nutritional balance required for long-term mushroom development. Although the addition of biochar to pure corn dust (T3–T5) slightly improved outcomes, yields remained significantly lower than other treatments.

The most remarkable results were obtained from mixed substrates. The 50S:50CD formulation (T6) significantly increased productivity, reaching 5.88 flushes and 85.36% BE. This confirms that combining rubber sawdust and corn dust creates an increasingly proportionate growth medium, likely improving overall composition and nutrient availability. Notably, the incorporation of slight to medium biochar levels into the mixed substrate further amplified performance. Treatments T7 (1% biochar) and T8 (5% biochar) achieved the highest biological efficiencies, exceeding 99% and peaking at 109.13%. These results indicate that moderately regulated biochar supplementation substantially improved substrate efficiency and mushroom productivity [32]. However, increasing biochar to 10% (T9) slightly reduced BE compared to 1–5% levels, though productivity remained significantly higher than non-mixed treatments. This suggests that while biochar is beneficial, excessive amounts might change nutritional balance or substrate systems [44].

Overall, the results demonstrate that the strategic integration of agro-waste materials with optimized biochar supplementation can markedly enhance mushroom yield, improve flush consistency, and increase substrate utilization efficiency. The 50S:50CD formulation supplemented with 1–5% biochar proved to be the most effective, delivering high productivity alongside sustainable waste valorization. These findings highlight the role of biochar as a valuable functional amendment, capable of converting agricultural residues into efficient, high-performance cultivation substrates while supporting circular and eco-efficient production systems. However, as this study was conducted under controlled conditions, further investigation is needed to confirm its applicability at a larger commercial scale and under varying environmental settings.

### 3.5. Nutritional Composition of Harvested Mushrooms

The proximate composition of *L. squarrosulus* fruiting bodies varied noticeably across substrate formulations, indicating that both agro-waste composition and biochar supplementation influenced not only yield but also nutritional quality (Table 6). Overall, all treatments produced dried fruiting bodies with high dry matter content (approximate 90.1–91.4%), corresponding to low moisture levels (approximate 8.5–9.9%). These values are consistent with those reported for many dried edible mushrooms (approximate 8.0–10.3%) [46] and indicate good post-harvest stability, making the products well-suited for storage and further processing into value-added products.

Crude protein content showed the most distinct variation among treatments. Mushrooms cultivated on 100% corn dust substrates (T2–T5) exhibited the highest protein levels, ranging from 31.77% to 35.90%, with T4 (100CD + 5% biochar) reaching the maximum value. This suggests that corn dust provides a more nitrogen-rich environment, which supports protein synthesis in fungal biomass [47]. In contrast, mixed substrates (T6–T9) produced moderate protein levels (approximate 22.70–25.01%), while 100% rubber sawdust (T1) resulted in lower protein content (19.24%), likely due to its relatively lower nutrient availability [48].

Crude fiber content followed an opposite trend. The highest fiber levels were recorded in T1 (29.05%) and in mixed substrate treatments (approximately 24–25%), particularly T8 (25.31%). This suggests that lignocellulosic-rich materials such as sawdust play a key role in enhancing structural fiber accumulation in mushrooms. The inclusion of sawdust in substrate formulations generally influenced fiber composition and, in some cases, contributed to a slight increase in crude fat content of *L. squarrosulus* fruiting bodies compared to non-sawdust substrates, consistent with findings reported by Osibe and Chiejina [49]. In contrast, corn dust-based treatments exhibited significantly lower fiber content (approximately 16–18%), reflecting differences in substrate composition and degradation patterns. Despite these variations, fat content remained consistently low across all treatments (approximately 1.0–1.96%), confirming that *L. squarrosulus* is inherently a low-fat food source regardless of substrate formulation. Ash content, representing total mineral composition, was slightly higher in biochar-amended treatments (T3–T5), reaching up to 6.62% in T4, suggesting that biochar may enhance mineral availability and uptake [50].

Carbohydrate content ranged from 29.75% to 37.53%, with the highest level observed in T7 (50S:50CD + 1% biochar). This suggests that mixed substrates promote a more balanced accumulation of carbohydrates, contributing to both energy value and desirable texture. Accordingly, the calculated energy values varied between 381.69 and 403.25 kcal/100 g, with the highest values recorded in biochar-amended corn dust treatments (T3 and T4). These findings indicate that substrate composition or cultivation conditions play a direct role in shaping carbohydrate distribution in both the fruiting bodies and the mycelial biomass [51].

Importantly, biochar supplementation at 1–5% appeared to enhance certain nutritional attributes, particularly protein and ash content, without negatively affecting other components. However, increasing biochar to 10% did not consistently improve nutritional quality, suggesting an optimal inclusion range. Overall, these findings demonstrate that substrate formulation plays a critical role in shaping the nutritional profile of mushrooms [50]. While corn dust-based substrates maximize protein content, mixed agro-waste formulations offer a more balanced composition of fiber, carbohydrates, and energy. This highlights an important opportunity to tailor mushroom nutritional quality through strategic substrate design, aligning production systems with both market demands and sustainable resource utilization.

### 3.6. Production Costs and Economic Profitability Analysis

The economic analysis clearly demonstrates that integrating corn dust with biochar substantially improved both production efficiency and profitability in mushroom cultivation (Table 7). While single-substrate approaches such as 100% sawdust (T1) and 100% corn dust (T2) provided baseline comparisons, their economic returns were limited. In particular, the corn dust-only treatment (T2) resulted in negative profit, indicating that low raw material cost alone does not guarantee economic sustainability when yields are poor. Although adding biochar into 100% corn dust (T3 and T5) helped improve yield slightly; however, higher biochar levels also increased input costs, which decreased overall returns.

In contrast, the combined substrate of 50% sawdust and 50% corn dust (T6) significantly enhanced productivity and economic performance. This balanced formulation improved yield per bag and generated substantially higher profits than single-material treatments. The synergy between the lignocellulosic composition of sawdust and the nutrient contribution of corn dust likely created a more favorable environment for mycelial growth and fruiting. Furthermore, the incorporation of biochar into this mixed substrate (T7–T9) further improved performance. Treatments supplemented with 1–5% biochar (T7 and T8) achieved the highest yields (0.134–0.138 kg per bag) and the greatest profits (13.29–13.63 THB per bag). These findings indicate that moderate biochar application enhances substrate functionality, particularly in terms of nutrient efficiency and substrate circulation, without substantially increasing production costs. From a practical perspective, the use of mixed substrates supplemented with 1–5% biochar represents an effective strategy for optimizing both yield and profitability in commercial mushroom cultivation. However, increasing biochar content to 10% led to a slight decline in profitability, likely due to higher input costs and diminishing returns in yield. This suggests that there is an optimal biochar threshold, beyond which economic benefits begin to decrease. 

Overall, the findings demonstrate that the strategic integration of agro-waste materials with moderate biochar supplementation provides a suitable compromise between production cost and productivity. Importantly, the results emphasize that lower-cost substrates alone do not guarantee higher economic returns; rather, achieving an appropriate balance between biological performance and input costs is essential. Among the tested formulations, the 50S:50CD substrate supplemented with 5% biochar proved to be the most economically efficient, delivering high yields while maintaining reasonable production expenses.

From an economic perspective, the findings clearly highlight that substrate formulation is a key determinant of both biological efficiency and overall profitability. More broadly, the conversion of locally available agricultural residues into value-added mushroom substrates not only enhances production performance but also strengthens farm-level economic returns. This approach promotes efficient resource utilization, minimizes waste, and supports more sustainable cultivation systems. By optimizing substrate composition, growers can shorten production cycles, increase yields, and improve profitability, positioning biochar-integrated agro-waste substrates as a practical and impactful innovation for modern mushroom cultivation.

### 3.7. Future Challenges and Opportunities for Development

Future development of mushroom substrates integrating biochar and agro-wastes presents both challenges and promising opportunities, particularly in optimizing cost-efficiency. One key challenge is reducing the reliance on supplementary ingredients (rice bran, calcium carbonate, calcium sulfate, and sodium sulfate), which, although beneficial for yield, contribute significantly to long-term production costs. Future research should explore whether biochar can partially substitute these additives while maintaining productivity. Another promising direction lies in fine-tuning the ratio between sawdust and corn dust, as current results suggest that proportioned mixtures enhance both productivity and nutrient availability. Adjusting these proportions may reduce the need for related supplements while preserving high biological efficiency. However, retaining this balance requires careful control of carbon-to-nitrogen ratios and combined substrate density. There is also an opportunity to utilize locally available agro-wastes to further reduce input costs and increase sustainability. Additionally, long-term cultivation trials are needed to evaluate consistency across multiple production cycles. Integrating these strategies could lead to more economically viable and environmentally sustainable mushroom cultivation systems.

## 4. Conclusions

This study clearly demonstrates that the strategic integration of agro-wastes and biochar can significantly enhance mushroom substrate performance, improving not only biological productivity but also economic viability. The results confirm that optimizing substrate balance is more critical than relying on a single low-cost material, as productivity and economic return are closely interconnected. Actually, a balanced formulation combining both materials creates a more favorable physicochemical environment, supporting efficient mycelial growth, reduced contamination, and accelerated production cycles. Moreover, biochar can serve as an effective additive to enhance substrate performance when applied at appropriate levels. Moderate supplementation levels (1–5%) proved particularly effective, enhancing mycelial colonization, increasing BE, and maximizing yield across multiple flushes of *L. squarrosulus*. While higher biochar levels (10%) still maintained strong performance, they did not consistently translate into additional benefits and may increase production costs, indicating the importance of optimization. Importantly, the 50:50 mixture of sawdust and corn dust enriched with 5% biochar emerged as the most promising formulation, achieving superior yield, high BE (>90%), and the highest yield and profitability. In addition to productivity gains, substrate design also influenced the nutritional quality of mushrooms, allowing for the potential tailoring of protein, fiber, and energy content through targeted formulation strategies. From an economic perspective, the integration of locally available agricultural residues with optimized biochar supplementation offers a practical solution to reduce waste, lower input costs, and enhance farm-level profitability. This approach provides valuable guidance for growers seeking to improve efficiency through sustainable substrate management, while aligning with circular economy principles by converting low-value biomass into high-value food products. All things considered, this study displays the potential of biochar-assisted agro-waste substrates as a sustainable innovation in mushroom cultivation. By optimizing substrate composition and reducing dependence on costly supplements, this strategy supports improved productivity, stronger economic outcomes, and greater environmental sustainability. Future research should focus on large-scale application and long-term performance under commercial production conditions. 

## Figures and Tables

**Table 1:** Formulations for the development of mushroom cultivation substrates through the integration of biochar and agro-industrial wastes, modified and improved from Zakil et al. [20].

Treatment Codes	Raw Substrates		Bamboo Biochar (%)	Remark
	Rubber Sawdust (%)	Corn Dust (%)		
T1-100S + SI	100	NA	NA	100 kg rubber sawdust + supplementary ingredients (no biochar)
T2-100CD + SI	NA	100	NA	100 kg corn dust + supplementary ingredients (no biochar)
T3-100CD + SI + 1BB-Char	NA	100	1	100 kg corn dust + supplementary ingredients + 1 kg bamboo biochar
T4-100CD + SI + 5BB-Char	NA	100	5	100 kg corn dust + supplementary ingredients + 5 kg bamboo biochar
T5-100CD + SI + 10BB-Char	NA	100	10	100 kg corn dust + supplementary ingredients + 10 kg bamboo biochar
T6-50S:50CD + SI	50	50	NA	50 kg rubber sawdust + 50 kg corn dust + supplementary ingredients (no biochar)
T7-50S:50CD + SI + 1BB-Char	50	50	1	50 kg rubber sawdust + 50 kg corn dust + supplementary ingredients + 1 kg bamboo biochar
T8-50S:50CD + SI + 5BB-Char	50	50	5	50 kg rubber sawdust + 50 kg corn dust + supplementary ingredients + 5 kg bamboo biochar
T9-50S:50CD + SI + 10BB-Char	50	50	10	50 kg rubber sawdust + 50 kg corn dust + supplementary ingredients + 10 kg bamboo biochar

**Note:** These additives have previously been reported as suitable supplements for cultivating mushrooms in the genera *Lentinus* and *Pleurotus* in Thailand [18]. NA indicates that the component was not included in the respective formulation.

**Table 3:** Radial mycelial growth and mycelial density of *L. squarrosulus* cultivated on different mixed substrate formulations.

Treatment	Radial Mycelial Growth (mm/Day)	Mycelial Density
T1-100S + SI	14.03 ± 0.16 ^de^	1.00 ± 0.00 ^c^
T2-100CD + SI	12.13 ± 0.53 ^f^	2.60 ± 0.55 ^b^
T3-100CD + SI + 1BB-Char	12.20 ± 0.34 ^f^	2.60 ± 0.55 ^b^
T4-100CD + SI + 5BB-Char	14.01 ± 0.34 ^de^	3.20 ± 0.45 ^ab^
T5-100CD + SI + 10BB-Char	14.55 ± 0.40 ^cd^	3.60 ± 0.55 ^a^
T6-50S:50CD + SI	13.88 ± 0.60 ^e^	2.60 ± 0.55 ^b^
T7-50S:50CD + SI + 1BB-Char	14.71 ± 0.62 ^c^	2.80 ± 0.45 ^b^
T8-50S:50CD + SI + 5BB-Char	16.19 ± 0.46 ^b^	3.60 ± 0.55 ^a^
T9-50S:50CD + SI + 10BB-Char	16.81 ± 0.33 ^a^	3.60 ± 0.55 ^a^

**Note:** Values are presented as mean ± standard deviation. Means within the same column followed by different letters are significantly different according to Duncan’s multiple range test (*p* < 0.05).

**Table 4:** Mycelial colonization of substrates, contamination rates in mushroom spawn, duration of primordia formation, and time to first harvest after bag opening.

Treatment	Completed Substrate Colonization (Days)	Contamination Rate (%)	Primordia Formation Period (Days)	First Harvestable Fruiting Bodies (Days)
T1-100S + SI	29.88 ± 1.74 ^c^	10.00	58.60 ± 8.64 ^d^	59.87 ± 8.75 ^d^
T2-100CD + SI	39.24 ± 2.74 ^a^	16.67	82.60 ± 10.89 ^a^	84.53 ± 11.12 ^a^
T3-100CD + SI + 1BB-Char	38.80 ± 1.47 ^a^	3.33	75.20 ± 10.78 ^b^	77.13 ± 11.04 ^b^
T4-100CD + SI + 5BB-Char	36.52 ± 1.71 ^b^	3.33	75.80 ± 9.97 ^b^	77.87 ± 10.25 ^b^
T5-100CD + SI + 10BB-Char	37.16 ± 1.82 ^b^	0.00	65.53 ± 12.08 ^c^	67.40 ± 12.34 ^c^
T6-50S:50CD + SI	29.80 ± 1.63 ^c^	10.00	49.20 ± 0.77 ^f^	51.33 ± 0.49 ^e^
T7-50S:50CD + SI + 1BB-Char	29.16 ± 1.34 ^c^	3.33	49.80 ± 1.37 ^f^	51.60 ± 0.51 ^e^
T8-50S:50CD + SI + 5BB-Char	27.64 ± 1.08 ^d^	0.00	52.07 ± 3.73 ^ef^	53.60 ± 1.68 ^ef^
T9-50S:50CD + SI + 10BB-Char	26.68 ± 1.52 ^d^	0.00	57.00 ± 2.36 ^de^	58.60 ± 2.26 ^de^

**Note:** Values are presented as mean ± standard deviation. Means within the same column followed by different letters are significantly different according to Duncan’s multiple range test (*p* < 0.05). The percentage contamination rate was determined by counting the number of contaminated mushroom bags among 30 prepared replicates and calculating the average.

**Table 5:** Number of flushes and biological efficiency measured in each substrate formulation throughout the cultivation period.

Treatment	Number of Flushes	BE (%)
T1-100S + SI	5.33 ± 0.87 ^a^	54.97 ± 13.47 ^c^
T2-100CD + SI	1.13 ± 1.08 ^c^	8.68 ± 2.60 ^d^
T3-100CD + SI + 1BB-Char	1.33 ± 1.09 ^bc^	11.29 ± 3.67 ^d^
T4-100CD + SI + 5BB-Char	0.83 ± 0.87 ^c^	6.85 ± 4.44 ^d^
T5-100CD + SI + 10BB-Char	2.04 ± 3.29 ^b^	19.41 ± 5.80 ^d^
T6-50S:50CD + SI	5.88 ± 0.34 ^a^	85.36 ± 20.35 ^b^
T7-50S:50CD + SI + 1BB-Char	5.50 ± 0.78 ^a^	109.13 ± 15.37 ^a^
T8-50S:50CD + SI + 5BB-Char	5.42 ± 0.72 ^a^	99.73 ± 27.15 ^a^
T9-50S:50CD + SI + 10BB-Char	5.17 ± 0.76 ^a^	84.67 ± 12.59 ^b^

**Note:** Values are presented as mean ± standard deviation. Means within the same column followed by different letters are significantly different according to Duncan’s multiple range test (*p* < 0.05).

**Table 6:** Proximate composition (%) and energy values (kcal/100 g) of dried *L. squarrosulus* fruiting bodies cultivated on different substrate formulations.

Treatment	Dry Mass (%)	Moisture (%)	Ash (%)	Crude Fiber (%)	Fat (%)	Crude Protein (%)	Carbohydrate (%)	Energy Value (kcal/100 g)
T1-100S + SI	90.27 ± 0.03	9.73 ± 0.03	4.54 ± 0.04	29.05 ± 0.27	1.01 ± 0.09	19.24 ± 0.58	36.43 ± 0.76	381.69 ± 0.95
T2-100CD + SI	90.11 ± 0.03	9.89 ± 0.03	5.75 ± 0.01	18.30 ± 0.06	1.33 ± 0.29	34.01 ± 0.04	30.73 ± 0.36	398.85 ± 2.09
T3-100CD + SI + 1BB-Char	90.47 ± 0.14	9.53 ± 0.14	6.02 ± 0.11	16.10 ± 0.88	1.66 ± 0.19	34.99 ± 0.11	31.70 ± 0.56	403.17 ± 0.25
T4-100CD + SI + 5BB-Char	90.61 ± 0.01	9.39 ± 0.01	6.62 ± 0.04	16.69 ± 0.33	1.65 ± 0.23	35.90 ± 0.17	29.75 ± 0.25	403.25 ± 1.38
T5-100CD + SI + 10BB-Char	90.51 ± 0.04	9.49 ± 0.04	5.85 ± 0.06	17.16 ± 0.92	1.39 ± 0.27	31.77 ± 0.59	34.34 ± 1.22	401.26 ± 0.80
T6-50S:50CD + SI	90.95 ± 0.07	9.05 ± 0.07	5.05 ± 0.04	24.10 ± 0.15	1.96 ± 0.17	23.97 ± 0.05	35.87 ± 0.26	392.07 ± 5.78
T7-50S:50CD + SI + 1BB-Char	91.17 ± 0.00	8.83 ± 0.00	5.21 ± 0.11	24.21 ± 0.33	1.52 ± 0.01	22.70 ± 0.84	37.53 ± 1.27	390.86 ± 1.16
T8-50S:50CD + SI + 5BB-Char	91.43 ± 0.05	8.57 ± 0.05	5.39 ± 0.05	25.31 ± 0.62	1.75 ± 0.20	24.60 ± 0.04	34.38 ± 0.86	389.87 ± 1.82
T9-50S:50CD + SI + 10BB-Char	91.23 ± 0.00	8.77 ± 0.00	5.23 ± 0.01	24.41 ± 0.47	1.79 ± 0.20	25.01 ± 0.43	34.79 ± 0.16	390.32 ± 1.53

**Note:** Values for each treatment are presented as mean ± standard deviation.

**Table 7:** Comparative analysis of production cost components and economic profitability among the developed mushroom cultivation substrate formulations.

Treatment	Main Substrates (THB per kg)		SI (kg) (8.2 THB/kg)	Biochar (15 THB/kg)				Mixture Cost per Bag (THB)	Overall Fix Cost per Bag (THB)	Total Cost per Bag (THB)	Yields (kg/Bag)	Selling Price (120 THB/kg) and Profitability (THB/bag)
	Sawdust (3 THB/kg)	Corn Dust (2 THB/kg)		0% (0 kg)	1% (1 kg)	5% (5 kg)	10% (10 kg)					
T1-100S + SI	100/ 300	NA/0	8.2/66.42	NA/0	NA/0	NA/0	NA/0	366.4/325 = 1.13	1.78	2.91	0.091	10.92–2.91 Profit = 8.01
T2-100CD + SI	NA/0	100/200	8.2/ 66.42	NA/0	NA/0	NA/0	NA/0	266.4/325 = 0.82	1.78	2.60	0.011	1.32–2.60 Profit = −1.28
T3-100CD + SI + 1BB-Char	NA/0	100/200	8.2/ 66.42	NA/0	1/15	NA/0	NA/0	281.4/328 = 0.86	1.78	2.64	0.016	1.92–2.64 Profit = −0.72
T4-100CD + SI + 5BB-Char	NA/0	100/200	8.2/ 66.42	NA/0	NA/0	5/75	NA/0	341.4/340 = 1.01	1.78	2.79	0.008	0.96–2.79 Profit = −1.83
T5-100CD + SI + 10BB-Char	NA/0	100/200	8.2/ 66.42	NA/0	NA/0	NA/0	10/150	416.4/355 = 1.17	1.78	2.95	0.022	2.64–2.95 Profit = −0.31
T6-50S:50CD + SI	50/150	50/100	8.2/ 66.42	NA/0	NA/0	NA/0	NA/0	316.4/325 = 0.98	1.78	2.75	0.116	13.92–2.75 Profit = 11.17
T7-50S:50CD + SI + 1BB-Char	50/150	50/100	8.2/ 66.42	NA/0	1/15	NA/0	NA/0	331.4/328 = 1.01	1.78	2.79	0.134	16.08–2.79 Profit = 13.29
T8-50S:50CD + SI + 5BB-Char	50/150	50/100	8.2/ 66.42	NA/0	NA/0	5/75	NA/0	391.4/340 = 1.15	1.78	2.93	0.138	16.56–2.93 Profit = 13.63
T9-50S:50CD + SI + 10BB-Char	50/150	50/100	8.2/ 66.42	NA/0	NA/0	NA/0	10/150	466.4/355 = 1.32	1.78	3.10	0.124	14.88–3.10 Profit = 11.78

**Note:** The initial cost estimation was calculated using 100 kg of raw materials, with sawdust priced at 2.5–3 THB/kg and corn dust at 1–2 THB/kg. The SI (8.2 THB/kg) included 5% rice bran, 1% calcium carbonate, 2% calcium sulfate, and 0.2% sodium sulfate (dry weight basis). Depending on the biochar level (8–15 THB/kg), the final substrate weight ranged from 108.2–118.2 kg. From this formulation, 1 kg of dry substrate produced approximately three mushroom bags. Each bag also incurred fixed costs: plastic bag (0.5 THB), tools (0.78 THB), and sterilization (0.5 THB), totaling about 1.78 THB per bag. Actual costs may vary by location.

## Data Availability

The original contributions presented in this study are included in the article. Further inquiries can be directed to the corresponding author.

## References

[1] Jayaraman S., Yadav B., Dalal R.C., Naorem A., Sinha N.K., Rao C.S., Dang Y.P., Patra A.K., Datta S.P., Rao A.S. (2024). Mushroom farming: A review focusing on soil health, nutritional security and environmental sustainability. Farming Syst..

[2] Patil S., Chonde S., Pathade G. (2024). Production of mushrooms: A short review. Ecol. Environ. Conserv..

[3] Liang C.H., Wu C.Y., Lu P.L., Kuo Y.C., Liang Z.C. (2019). Biological efficiency and nutritional value of the culinary-medicinal mushroom *Auricularia* cultivated on a sawdust basal substrate supplement with different proportions of grass plants. Saudi J. Biol. Sci..

[4] Aa B., Ab O., Ns Y. (2024). Mushroom cultivation in tropical Africa: Successes, challenges, and opportunities. J. Agric. Food Res..

[5] Ojha R.K., Fayaz A., Kaundal M. (2025). Sustainable substrates for mushroom production: A review. J. Adv. Biol. Biotechnol..

[6] Kamthan R., Tiwari I. (2017). Agricultural wastes-potential substrates for mushroom cultivation. Eur. J. Exp. Biol..

[7] Aiduang W., Jatuwong K., Jinanukul P., Suwannarach N., Kumla J., Thamjaree W., Teeraphantuvat T., Waroonkun T., Oranratmanee R., Lumyong S. (2024). Sustainable innovation: Fabrication and characterization of mycelium-based green composites for modern interior materials using agro-industrial wastes and different species of fungi. Polymers.

[8] Maqsood S., Navaf M., Kumar P., Yücetepe A., Thuy N.N.T., Ozkan G., Moreno A., Capanoglu E., Khalid W., Esatbeyoglu T. (2025). Sustainable utilization of corn waste and their role toward the circular economy. J. Agric. Food Res..

[9] Mattayaruk T., Yangngam Y., Cheas S., Suntara C., Wanapat M., Supapong C., Lunpha A., Pilajun R., Intawicha P., Cherdthong A. (2026). Effects of NSP Enzymes and *Candida tropicalis* KKU20 on the Nutritional and Fermentation Characteristics of Corn Dust. Fermentation.

[10] Mihilall Y., Mudhoo A., Mohee R. (2011). Development of a new substrate for the cultivation of the *Pleurotus sajor-caju* mushroom through controlled composting. Dyn. Soil Dyn. Plant.

[11] Dong H.R., Jiang N., Zhang D., Li Y., Zhou F., Li Z.P., Li Q.Z., Tan Q., Zhang M.Y., Yu H.L. (2025). Research progress and prospect of substrate alternatives for edible fungi based on the “cycle production of plants, animals, and fungi”. J. Fungi.

[12] Zhang Y., Chen H., Islam S. (2025). Advances in biochar modification for environmental remediation with emphasis on iron functionalization. Biochar X.

[13] Hu W., Di Q., Liang T., Liu J., Zhang J. (2022). Effects of spent mushroom substrate biochar on growth of oyster mushroom (*Pleurotus ostreatus*). Environ. Technol. Innov..

[14] Chaturvedi K., Singhwane A., Dhangar M., Mili M., Gorhae N., Naik A., Prashant N., Srivastava A.K., Verma S. (2024). Bamboo for producing charcoal and biochar for versatile applications. Biomass Convers. Biorefin..

[15] Mushroomsubstrate How to Improve the Porosity of Your Mushroom Substrate. https://www.mushroomsubstrate.com/blogs/mushroom-substrate-blog/improve-mushroom-substrate-porosity?srsltid=AfmBOorPaf6CtM8fb-hRI9pnY4ybjcPgurhM2Z4nQHsxjZBJG4ZdzELK.

[16] Karlsson M., Jönsson H.L., Hultberg M. (2025). Inclusion of biochar in mushroom substrate influences microbial community composition of the substrate and elemental composition of the fruiting bodies. Sci. Total Environ..

[17] Lamichhane S., Wangchuk D., Aye T. (2025). A comparative research on the effect of biochar supplementation on growth and yield of *Pleurotus ostreatus*. Int. J. Adv. Acad. Stud..

[18] Kupradi C., Khongla C., Musika S., Ranok A., Tamaruay K., Woraratphoka J., Mangkalanan S. (2017). Cultivation of *Lentinus squarrosulus* and *Pleurotus ostreatus* on cassava bagasse based substrates. Int. J. Agric. Technol..

[19] Mudakir I., Hastuti U.S. (2015). Study of wood sawdust with addition of plantation wastes as a growth medium on yields and quality of white oyster mushroom. Agrivita J. Agric. Sci..

[20] Zakil F.A., Sueb M.S.M., Isha R., Kamaluddin S.H. (2021). Efficiency of charcoal as supporting growth material in *Pleurotus ostreatus* mushroom cultivation on various agricultural wastes mixed with rubber tree sawdust (SR). Chem. Eng. Trans..

[21] Bhattarai R., Karki N., Shakya S., Dhakal R.P., Poudel P. (2024). Potential application of biochar as a growth supplement for mushroom cultivation (*Pleurotus ostreatus*). Int. J. Hortic. Food Sci..

[22] Lopez M., Olsen H., Khalil A. (2025). Nutritional enhancement of oyster mushrooms using biochar-enriched substrates: Implications for household consumption. Int. J. Home Sci..

[23] Sangsuwan P., Detraksa J., Srimawong P. (2023). Proximate analysis of the growth of organic grey oyster mushrooms on biochar from agricultural waste. Pak. J. Phytopathol..

[24] Torres E., Müller I., García C. (2025). Economic feasibility of biochar as a low-cost growth supplement in oyster mushroom production. Int. J. Financ. Manag. Econ..

[25] Psurtseva N.V., Kiyashko A.A., Senik S.V., Shakhova N.V., Belova N.V. (2023). The conservation and study of macromycetes in the Komarov Botanical Institute Basidiomycetes Culture Collection—Their taxonomical diversity and biotechnological prospects. J. Fungi.

[26] Aiduang W., Patipattanakul T., Keduk Y., Rattanapat A., Phumila P., Jinanukul P., Sysouphanthong P., Xayyavong O., Jatuwong K., Lumyong S. (2025). Looking at the possibility of using mushroom mycelium for developing leather-like materials aligned with eco-friendly and sustainable fashion trends. Life.

[27] Jatuwong K., Aiduang W., Kiatsiriroat T., Kamopas W., Lumyong S. (2024). Effects of biochar and arbuscular mycorrhizal fungi on soil health in Chinese kale (*Brassica oleracea* var. *alboglabra* L.) cultivation. Microbiol. Res..

[28] Kalra Y.P., Maynard D.G. (1991). Methods Manual for Forest Soil and Plant Analysis.

[29] De Leon A.M., Guinto L.J.Z.G., De Ramos P.D.V., Kalaw S.P. (2017). Enriched cultivation of *Lentinus squarrosulus* (Mont.) Singer: A newly domesticated wild edible mushroom in the Philippines. Mycosphere.

[30] Hussein J.M., Tibuhwa D.D., Mshandete A.M., Kivaisi A.K. (2016). Successful domestication of *Lentinus sajor-caju* from an indigenous forest in Tanzania. J. Appl. Biosci..

[31] Chiejina N.V., Osibe D.A. (2015). Oil palm fruit fibre promotes the yield and quality of *Lentinus squarrosulus* (Mont.) Singer, an edible Nigerian mushroom. Afr. J. Biotechnol..

[32] Nuralykyzy B., Nie J., Mei H., Zhang Y., Rogers K.M., Li C., Yuan Y. (2025). Synergies between carbon sequestration, nitrogen utilization, and mushroom quality: A comprehensive review of substrate, fungi, and soil interactions. J. Agric. Food Chem..

[33] Melanouri E.M., Diamantis I., Dedousi M., Dalaka E., Antonopoulou P., Papanikolaou S., Politis I., Theodorou G., Diamantopoulou P. (2025). *Pleurotus ostreatus*: Nutritional enhancement and antioxidant activity improvement through cultivation on spent mushroom substrate and roots of leafy vegetables. Fermentation.

[34] Chu J.N., Young C.C., Tan C.C., Wu S.P., Young L.S. (2012). Improvement of productivity and polysaccharide-protein complex in *Agaricus blazei Pesqui*. Agropecu. Bras..

[35] Krupodorova T.A., Barshteyn V.Y., Sekan A. (2021). Review of the basic cultivation conditions influence on the growth of basidiomycetes. Curr. Res. Environ. Appl. Mycol..

[36] Chen D., Yu X., Song C., Pang X., Huang J., Li Y. (2016). Effect of pyrolysis temperature on the chemical oxidation stability of bamboo biochar. Bioresour. Technol..

[37] Chutimanukul P., Phetkaew P., Sukdee S., Thepsilvisut O., Ehara H. (2025). Comparison of growth, yield, and carbon dioxide emission after cultivation of five edible mushrooms. Resources.

[38] Hoa H.T., Wang C.L., Wang C.H. (2015). The effects of different substrates on the growth, yield, and nutritional composition of two oyster mushrooms (*Pleurotus ostreatus* and *Pleurotus cystidiosus*). Mycobiology.

[39] Onyeka E.U., Udeogu E., Umelo C., Okehie M.A. (2018). Effect of substrate media on growth, yield and nutritional composition of domestically grown oyster mushroom (*Pleurotus ostreatus*). Afr. J. Plant Sci..

[40] Bereket K., Tesfaye B., Tadesse B., Tesfaw A. (2025). Optimizing growth, yield, and antioxidant properties of *Pleurotus ostreatus* M2191 and *Pleurotus sajor-caju* M2345 using industrial and agricultural waste substrates. Clean. Circ. Bioecon..

[41] Hu W., Gou L., Hu L., Wang S., Liang T., Zhou N. (2025). Effect of acid modification of biochar derived from spent mushroom substrate on the production of oyster mushroom (*Pleurotus ostreatus*). Sci. Rep..

[42] Meyer D., Oliveira M., Gutierrez A. (2025). Adoption potential of biochar supplementation in small-scale oyster mushroom farming: A social and extension perspective. Int. J. Agric. Ext. Soc. Dev..

[43] Lombardi N., Pironti A., Manganiello G., Marra R., Vinale F., Vitale S., Lorito M., Woo S.L. (2023). *Trichoderma* species problematic to the commercial production of *Pleurotus* in Italy: Characterization, identification, and methods of control. Microbiol. Res..

[44] Thakuri A., Kharel S., Maharjan R. (2025). Influence of low-dose biochar supplementation on yield and growth characteristics of oyster mushroom (*Pleurotus ostreatus*). Int. J. Agric. Food Sci..

[45] Tasnim F., Chowdhury A., Jahan N. (2025). Cost-benefit analysis of using biochar as a substrate supplement in commercial mushroom cultivation (*Pleurotus ostreatus*). Int. J. Res. Manag..

[46] Akata I., Ergonul B., Kalyoncu F. (2012). Chemical compositions and antioxidant activities of 16 wild edible mushroom species grown in Anatolia. Int. J. Pharmacol..

[47] Carrasco J., Zied D.C., Pardo J.E., Preston G.M., Pardo-Giménez A. (2018). Supplementation in mushroom crops and its impact on yield and quality. AMB Express.

[48] Rhaman S.M.S.A., Naher L. (2022). Evaluating carbon, nitrogen and heavy metal content in different agriculture biomass for mushroom substrate. International Conference on Bioengineering and Technology (IConBET2021), Kelantan, Malaysia, 24–25 May 2021.

[49] Osibe D.A., Chiejina N.V. (2015). Assessment of palm press fibre and sawdust-based substrate formulas for efficient carpophore production of *Lentinus squarrosulus* (Mont.) Singer. Mycobiology.

[50] Elkanah F.A., Oke M.A., Adebayo E.A. (2022). Substrate composition effect on the nutritional quality of *Pleurotus ostreatus* (MK751847) fruiting body. Heliyon.

[51] Araújo-Rodrigues H., Amorim M., de Freitas V., Relvas J.B., Tavaria F.K., Pintado M. (2025). Comparative analysis of polysaccharide and nutritional composition of biological and industrial-scale cultivated *Pleurotus ostreatus* mushrooms for functional food and nutraceutical applications. Polysaccharides.

